# Impact of Postoperative Antithrombotic Treatment on Patency and Survival After Open Posterior Popliteal Artery Aneurysm Repair: A Multicenter Retrospective Cohort Analysis from the PARADE Study

**DOI:** 10.3390/jcm15145364

**Published:** 2026-07-09

**Authors:** Giorgio Prouse, Giulia Bertagna, Francesco Andreoli, Maria Antonella Ruffino, Valentina Scarati, Raffaella Berchiolli, Stefanie Hayoz, Mariacarla Andreozzi, Alessandro Robaldo, Nicola Troisi

**Affiliations:** 1 Division of Vascular Surgery and Angiology, Centro Vascolare Ticino, Ente Ospedaliero Cantonale, CH-6900 Lugano, Switzerland; francesco.andreolimd@gmail.com (F.A.); mariacarla.andreozzi@eoc.ch (M.A.); alessandro.robaldo@eoc.ch (A.R.); 2 Vascular Surgery Unit, Department of Translational Research and New Technologies in Medicine and Surgery, University of Pisa, I-56126 Pisa, Italy; giuliaberty.it@hotmail.it (G.B.); valentinascarati@gmail.com (V.S.); raffaella.berchiolli@unipi.it (R.B.); nicola.troisi@unipi.it (N.T.); 3 Imaging Institute of Southern Switzerland (IIMSI), Ente Ospedaliero Cantonale, CH-6900 Lugano, Switzerland; mariaantonella.ruffino@eoc.ch; 4 Faculty of Biomedical Science, Università della Svizzera Italiana USI, CH-6900 Lugano, Switzerland; 5 Swiss Cancer Institute, CH-3008 Bern, Switzerland; stefanie.hayoz@sakk.ch

**Keywords:** popliteal artery aneurysm, posterior approach, anticoagulant therapy, antithrombotic therapy, antiplatelet therapy

## Abstract

**Background/Objectives**: This study assessed the impact of different postoperative antithrombotic strategies on graft patency and overall survival after open posterior repair of popliteal artery aneurysms (PAAs). **Methods**: A retrospectively maintained dataset of consecutive PAAs electively treated in 40 centers between January 2010 and December 2023 was analyzed. The study included patients undergoing elective open posterior repair. Primary outcomes were primary patency and overall survival according to postoperative antiplatelet or anticoagulant therapy. Secondary outcomes included secondary patency and major adverse cardiovascular events (MACEs). Kaplan–Meier analysis, log-rank testing, and Cox regression models were applied. **Results**: Overall, 638 patients were included. The cohort was predominantly male (96%), with a median age of 70 years. At 30 days, one death (0.2%) and seven MACEs (1.1%) occurred. Postoperative therapy was not associated with 30-day primary patency or MACEs. At a median follow-up of 30 months, overall survival was 90.3%. Kaplan–Meier analysis showed comparable overall survival across antithrombotic regimens, with a significant difference observed only between patients receiving combined anticoagulant and antiplatelet therapy and those receiving other regimens (*p* = 0.018). No differences emerged between vitamin K antagonists (VKAs) and direct oral anticoagulants (DOACs), nor between single or dual antiplatelet therapy and anticoagulation. Multivariable Cox regression showed that combined therapy was associated with poorer overall survival compared with other postoperative antithrombotic regimens (CT vs. others: HR of 1.30). Primary and secondary patency were also similar among regimens except for combined therapy (primary patency: *p* = 0.004; secondary patency: *p* = 0.018). **Conclusions**: Postoperative medical therapy was not associated with 30-day outcomes. During long-term follow-up, overall survival and graft patency were lower in patients receiving combined anticoagulant and antiplatelet therapy. Bleeding outcomes were not captured in this registry; as bleeding may contribute to the worse outcomes observed with combined therapy, this represents an important limitation.

## 1. Introduction

Popliteal artery aneurysms (PAAs) are the most common peripheral arterial aneurysms. Because of their complication risk, Society for Vascular Surgery (SVS) guidelines recommend treating asymptomatic PAAs > 20 mm [1]. When anatomically suitable, open posterior repair remains a preferred approach with excellent outcomes [2,3,4]. However, optimal postoperative medical therapy is not well established. Current European and North American peripheral arterial disease (PAD) guidelines favor single antiplatelet therapy, with intensified antithrombotic regimens reserved for selected patients [5,6,7,8]. Recent trials indicate potential benefit of stronger antithrombotic strategies in peripheral arterial occlusive disease [7,8,9,10]. Moreover, patients with PAAs have higher rates of atrial fibrillation and other indications for therapeutic anticoagulation [11,12]. Yet evidence guiding postoperative therapy after PAA repair is sparse and largely extrapolated from PAD revascularization studies [13,14,15]. Importantly, PAA patients differ from typical PAD bypass populations, and no randomized trial has specifically evaluated antithrombotic therapy after open PAA repair; available data come mainly from retrospective series and registries [15,16,17]. Anatomical and technical factors, including conduit type and distal runoff, also influence graft performance, but their interaction with postoperative antithrombotic therapy remains poorly defined. This study aimed to assess the impact of postoperative antithrombotic strategies on graft patency and overall survival after open posterior PAA repair, with stratification by runoff status and graft type.

## 2. Materials and Methods

### 2.1. Study Design and Patient Population

A multicenter retrospective cohort study was conducted under the auspices of the Research Collaborative in Peripheral Arterial Disease, a pan-European scientific network of vascular specialists (RCPAD; https://www.rcpad.org (accessed on 24 January 2026)). A total of 40 departments across 10 countries participated. Each center maintained its own registry, in which patient data were recorded at the time of surgery and during follow-up, and subsequently aggregated into the PARADE database (elective surgical repair of Popliteal ARtery Aneurysms with posterior approach vs. enDovascular Exclusion) [18].

The present study is a secondary analysis of the PARADE database, focusing on postoperative antithrombotic and anticoagulant therapy in the subgroup of patients electively treated via surgical posterior approach for exclusion of a PAA. Preoperative duplex ultrasound (DUS) and computed tomography angiography (CTA) were required for inclusion in both the registry and the present analysis. Owing to the pragmatic study design, all participating departments followed their routine local or regional protocols for perioperative medication and follow-up assessment. The minimum recommended follow-up consisted of physical examination combined with DUS or CTA at 1 month after the index procedure, at 6 months, and annually thereafter. All patients provided written informed consent for the procedure and for the use of their data in anonymized form. This study represents a secondary analysis of existing clinical data that were fully anonymized prior to inclusion in the multicenter database. In accordance with applicable local regulations at the participating centers, the secondary use of anonymized retrospective data for research purposes did not require additional ethics committee oversight. The study posed no additional risk to participants.

Within the registry, the 40 participating treating centers retrospectively identified patients with PAA who underwent elective treatment between January 2010 and December 2023. Local site investigators identified all consecutive patients meeting the inclusion criteria who had undergone preoperative DUS and CTA, and the corresponding data were retrospectively extracted from the anonymized database. Over the 14-year study period, 971 consecutive patients with PAA were treated at the participating centers, either by open posterior approach or by endovascular exclusion using covered stents.

**Figure 1:**
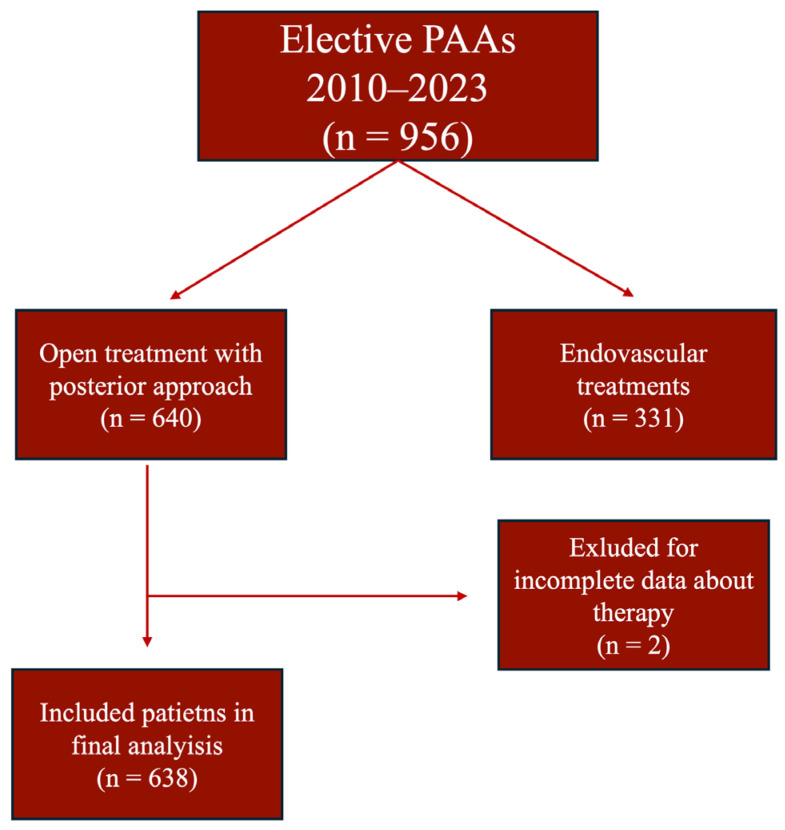
Study flow diagram showing the final cohort of analyzed patients. Abbreviations: PAAs, popliteal artery aneurysms.

For the present analysis, patients who underwent endovascular treatment were excluded. Of the initial cohort of 971 patients, 640 met the inclusion criteria. An additional two patients were excluded because of incomplete data. The study inclusion flowchart is shown in Figure 1.

### 2.2. Pre-Operative Assessment and Surgical Procedure

All patients underwent a standardized preoperative evaluation including clinical examination, DUS, and CTA to determine the length of the aneurysmal segment and to measure popliteal artery diameters 1 cm proximal and 1 cm distal to the aneurysmal sac. Bypass length was measured preoperatively on CTA using axial and coronal multiplanar reconstructions or centerline analysis, defined as the distance from the proximal to the distal anastomotic site. Intraoperatively, all patients received systemic heparinization, administered either as a weight-based dose (80 IU/kg) or as a fixed dose of 5000 IU, according to institutional preference. At the end of the procedure, heparin reversal with protamine was routinely performed at some centers, whereas others did not use protamine.

### 2.3. Definitions and Outcomes

All procedural data were retrospectively collected in a dedicated database. The dataset included patient demographics, preoperative risk factors, intraoperative details, 30-day outcomes, and follow-up information. All reinterventions were recorded in the same database and independently reviewed by the principal investigator and site collaborators. Data were pseudonymized at the treating centers prior to transfer and stored in a fully anonymized form within the multicenter database.

Chronic limb-threatening ischemia was defined according to global guidelines [6] and SVS reporting standard for chronic lower extremity peripheral artery disease [19].

Runoff status was assessed based on the number of patent tibial arteries (one-, two-, or three-vessel runoff), as determined by imaging obtained during the index procedure (diagnostic angiography) and preoperative DUS or CTA. A vessel was considered patent if continuous flow was demonstrated to the ankle level. Tibial vessels with distal occlusion below the ankle were not counted as patent runoff vessels.

Major cardiovascular adverse events (MACEs) were defined as a composite of cardiovascular death, myocardial infarction (MI), and stroke according to the SVS reporting standard [7,19]. Conduit type was categorized as autologous vein (great saphenous vein, small saphenous vein, or arm veins), prosthetic graft, or homograft.

The primary outcomes were primary patency and overall survival (defined as patient survival from the index procedure to death from any cause, irrespective of graft status) during follow-up, stratified according to postoperative antiplatelet or anticoagulant therapy. The number of runoff vessels, conduit type, and bypass length were included as covariates to evaluate their impact on the primary outcomes. Secondary outcomes included secondary patency and the incidence of 30-day MACEs, mortality and re-intervention, according to postoperative therapy.

Primary patency was defined on the basis of current reporting standard [19] as uninterrupted patency of the bypass graft with no interventions performed on or at the margins of the graft. Secondary patency was defined as patency maintained or restored by any secondary intervention (including thrombectomy, thrombolysis, or surgical revision) [19].

Graft occlusion was assessed through DUS or CTA depending on the participating centers and specific setting.

All other clinical and imaging definitions followed the reporting standards of the Society for Vascular Surgery for peripheral artery disease [19].

### 2.4. Antithrombotic Therapy

Postoperative antithrombotic therapy was left to the discretion of the treating physician and was classified as single antiplatelet therapy (SAPT), dual antiplatelet therapy (DAPT), oral anticoagulation (vitamin K antagonist or direct oral anticoagulant), or combinations thereof. Individual antithrombotic agents were also analyzed separately. The PARADE registry did not systematically capture the specific clinical rationale underlying therapy selection, including conditions such as atrial fibrillation, recent coronary interventions, hypercoagulable states, or perceived thrombotic risk related to graft or runoff characteristics.

### 2.5. Statistical Analysis

The distribution of continuous variables was assessed using the Shapiro–Wilk test. Normally distributed variables are presented as mean ± standard deviation (SD) and were compared using Student’s *t* test. Non-normally distributed variables are reported as median and interquartile range (IQR), with group differences assessed using the Mann–Whitney U test. Categorical variables are expressed as counts and percentages and were compared using the chi-square test or Fisher’s exact test, as appropriate.

Survival and primary patency were estimated using the Kaplan–Meier method, with comparisons performed using the log-rank test. To identify independent factors associated with primary outcomes, Cox proportional-hazard regression models were constructed. Variables included in multivariable analyses were selected based on clinical relevance and predefined covariates of interest. Hazard ratios (HRs) with 95% confidence intervals (CIs) are reported. Goodness of fit was evaluated using C-index and AIC. Proportional-hazard assumptions were checked using the Grambsch–Therneau test and Schoenfeld residuals.

Missing data were handled using available-case analysis. No formal multiple imputation procedure was performed because the proportion of missing covariate data was limited. Complete-case analyses were applied where appropriate depending on variable availability within each model. All the patients were censored at the last available follow-up and the median follow up as well as follow-up index was calculated. Missing data were minimal for the main study variables: baseline demographic and clinical characteristics were complete in all 638 patients, while among intraoperative variables graft material was missing in 5 cases (0.8%), conduit subtype in 4 cases (1.3%), and prosthetic graft subtype in 1 case (0.3%); preoperative and postoperative antithrombotic therapy were recorded in all patients.

Given that death may preclude the occurrence of loss of primary patency, a competing risk analysis was performed using the Fine–Gray subdistribution hazard model, treating death as a competing event. Subdistribution hazard ratios (sHRs) with 95% confidence intervals were calculated to assess the effect of therapy on primary patency.

Baseline imbalances between treatment groups were addressed using inverse probability of treatment weighting (IPTW) based on propensity scores derived from clinically relevant covariates (smoking history, CAD history (including AF), graft materials, bypass length, runoff BTK, pre-operative therapy). Treatment effects on overall survival and primary patency were estimated using weighted Cox proportional-hazard models with robust standard errors. Given the retrospective observational multicenter nature of the registry, all analyses should be considered exploratory and hypothesis-generating. Statistical significance was defined as a two-sided *p* value < 0.05. All statistical analyses were performed using R version 4.5.0 (R Foundation for Statistical Computing, Vienna, Austria).

## 3. Results

### 3.1. Baseline, Demographics and Intraoperative Details

A total of 956 elective PAAs were initially recorded in the registry. After applying the inclusion criteria, 638 patients were selected for the present analysis (Figure 1).

The cohort was predominantly male (n = 615, 96%), with a median age of 70 years (IQR: 64–77). At presentation, 68.4% were asymptomatic, 18.0% reported intermittent claudication, and 13.6% had chronic limb-threatening ischemia. Hypertension (75.6%), hyperlipidemia (60.3%), and a history of smoking (70.5%) were the most frequent comorbidities.

Regarding antithrombotic therapy, 404 patients (63.3%) were on SAPT (338 on ASA 100 mg daily, 57 on clopidogrel 75 mg daily, 6 on dipyridamole 250 mg twice daily, and 3 on ticagrelor 90 mg twice daily). DAPT was used by 6.3% of patients, while 5% were on vitamin K antagonists (VKAs) and 6.4% on full-dose direct oral anticoagulants (DOACs). Forty-nine patients (7.7%) were on a combination of antiplatelet and anticoagulant therapy (combined therapy, CT), and 11.3% were not receiving any antiplatelet or anticoagulant treatment. Additional baseline characteristics of the study population are reported in Table 1.

Autologous vein grafts were used in 46.5% of patients, prosthetic conduits in 50.9%, and homografts in 2.5%. Among patients treated with autologous grafts, the ipsilateral great saphenous vein was the most used conduit.

The median bypass length was 65 mm (IQR: 50–100), and the median graft diameter was 7 mm (IQR: 5–8). Technical success was achieved in 99.4% of procedures, with failures related to acute occlusions. Adjunctive intraoperative procedures were required in 8.8% of cases, most frequently below-knee thrombectomy. Intraoperative details are summarized in Table 2.

### 3.2. Early Outcomes

Postoperative medical management is summarized in Table 3.

Early major adverse cardiovascular events (MACEs) occurred in seven patients (1.1%). Comparing all the possible antithrombotic regimens, MACE rate was higher in patients under DAPT (*p* = 0.026), with a non-significant association with MACEs in the multivariable logistic regression model (*p* = 0.144); the full model is reported in Table S1. No differences between patients under CT vs. all other (*p* = 0.3) regimens were registered. Early graft occlusion was observed in 13 patients (2.0%), and procedure-related reinterventions were required in 25 cases (3.9%).

In the multivariable logistic regression analysis, postoperative therapy showed no significant association with 30-day primary patency or re-intervention. The full model is provided in Table S2.

### 3.3. Long-Term Overall Survival

At a median follow-up of 30 months (IQR: 8–45), overall survival was 90.3%. The most common cause of death were acute MI and cancer (9/62, 14%) with a consistent number of deaths with unknown causes (21/62, 33.9%) Details about death causes are reported in Table S3. The median follow-up index was 0.65 [0.39–0.91].

**Figure 2:**
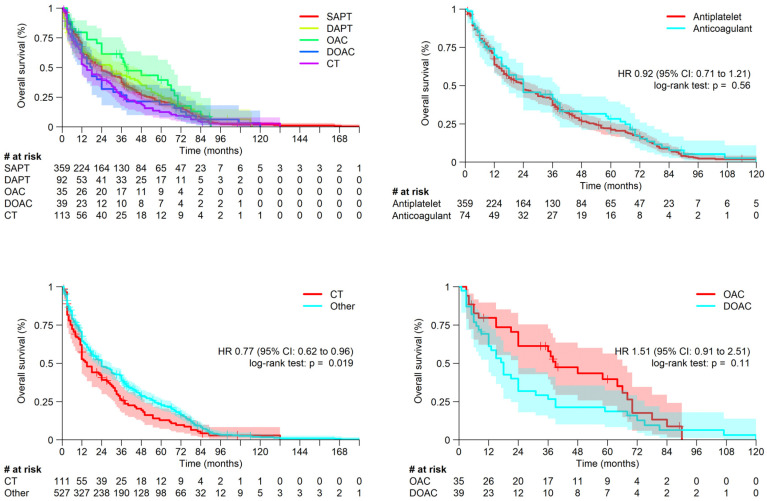
Kaplan–Meier curves for overall survival by post-operative antithrombotic therapy. Abbreviations: #, number; CI, confidence interval; CT, combined therapy (anticoagulant + SAPT); DAPT, double antiplatelet therapy; DOAC, direct oral anticoagulant; HR, hazard ratio; SAPT, single antiplatelet therapy; VKA, vitamin K antagonist.

Kaplan–Meier analysis (Figure 2) demonstrated comparable overall survival across the different postoperative antithrombotic strategies, with the only significant difference observed between patients receiving CT and all other regimens. No significant differences were found between VKA and DOAC, nor between antiplatelet therapy (SAPT or DAPT) and anticoagulation (VKA or DOAC).

According to the multivariable Cox regression model (Table 4), patients receiving combined therapy (CT) experienced worse overall survival compared with patients receiving other postoperative antithrombotic regimens. (CT vs. others: HR of 1.30, 95% CI of 1.04–1.61; C = 0.523; AIC = 6312) Baseline characteristics of patients receiving CT versus all other regimens are reported in Supplementary Table S4. The median length of follow-up as well as the median follow-up index was not significantly different between groups (*p* = 0.239 and *p* = 0.0627 respectively).

### 3.4. Long-Term Patency

During follow-up, overall primary patency was 86.1%. Seventy-eight (10.8%) patients required late reintervention for a cumulative secondary patency of 94.7%.

**Figure 3:**
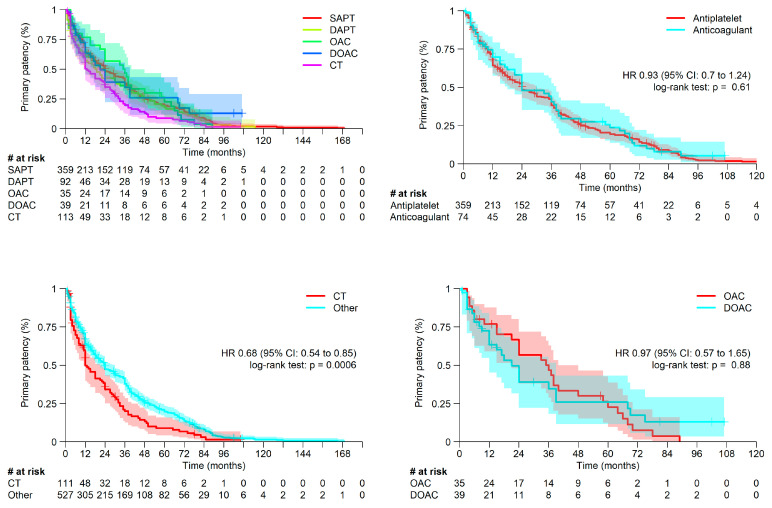
Kaplan–Meier curves for primary patency by antithrombotic therapy. Abbreviations: #, number; CI, confidence interval; CT, combined therapy (anticoagulant + SAPT); DAPT, double antiplatelet therapy; DOAC, direct oral anticoagulant; HR, hazard ratio; SAPT, single antiplatelet therapy; VKA, vitamin K antagonist.

Kaplan–Meier curves (Figure 3) showed primary patency to be similar between all antithrombotic regimens except for CT versus all other therapies. Furthermore, no differences in terms of primary patency were noted comparing VKA and DOAC as well as antiplatelet versus anticoagulant therapy (VKA and DOAC). The multivariable Cox analysis reported that CT was associated with a 47% increase in the hazard of primary patency loss compared with all other therapies and demonstrated that bypasses longer than 75 mm were associated with an increased risk of primary patency failure (HR of 1.41). The full model is shown in Table 5. To account for the competing risk of death, a competing risk analysis was performed using a Fine–Gray subdistribution hazard model, treating death as a competing event for primary patency (Supplementary Figure S1). In this analysis, CT remained independently associated with a significantly increased risk of primary patency loss compared with other postoperative antithrombotic regimens (CT vs. others: sHR of 1.28, 95% CI of 1.06–1.56; *p* = 0.011, C = 0.53, AIC = 5972).

Similarly the cumulative incidence function showed a significant difference in primary patency loss between treatment groups (*p* = 0.024), whereas no significant differences were observed for the competing event of death (*p* = 0.635) (Figure S1).

**Figure 4:**
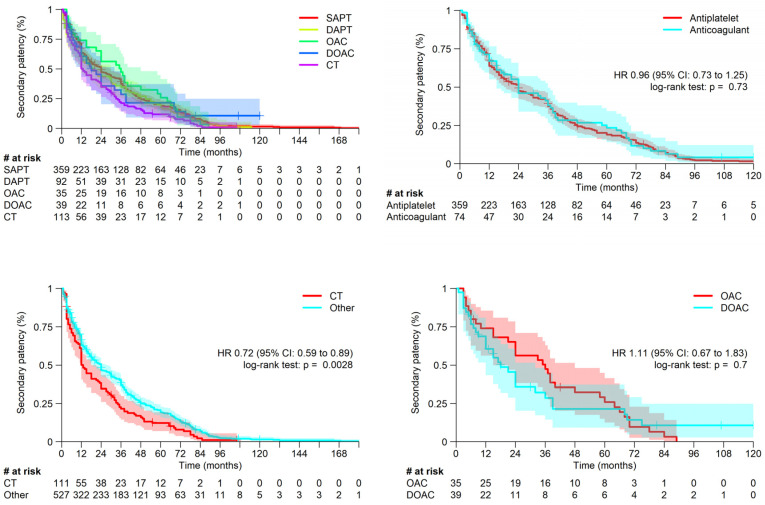
Kaplan–Meier curves for secondary patency by antithrombotic therapy. Abbreviations: #, number; CI, confidence interval; CT, combined therapy (anticoagulant + SAPT); DAPT, double antiplatelet therapy; DOAC, direct oral anticoagulant; HR, hazard ratio; SAPT, single antiplatelet therapy; VKA, vitamin K antagonist.

Similar results were obtained for secondary patency (Figure 4); patients under CT experienced a lower secondary patency compared to all other antithrombotic regimens. According to the Cox model, CT was associated with a 35% increase in the hazard of secondary patency failure compared to other therapies, and the usage of a vein conduit increased the estimated secondary patency over time of 18% (HR of 1.18, *p* = 0.045). The full model is reported in Table 6.

### 3.5. Inverse Probability of Treatment Weighting Analysis

**Figure 5:**
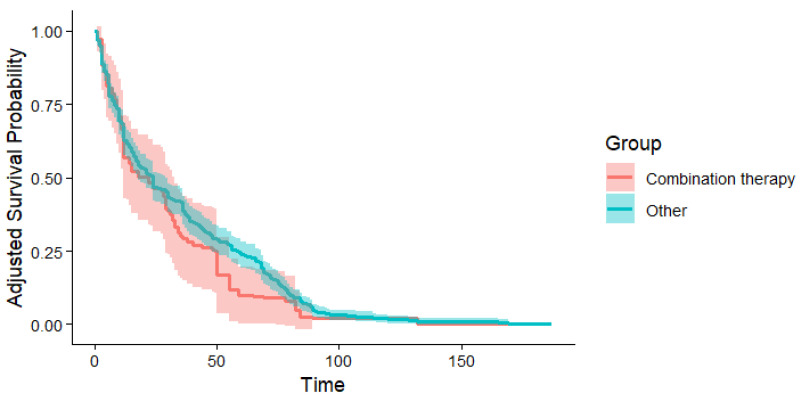
Kaplan–Meier estimates of overall survival after inverse probability of treatment weighting (IPTW) comparing patients treated with combined therapy (CT) and non-combined therapy.

**Figure 6:**
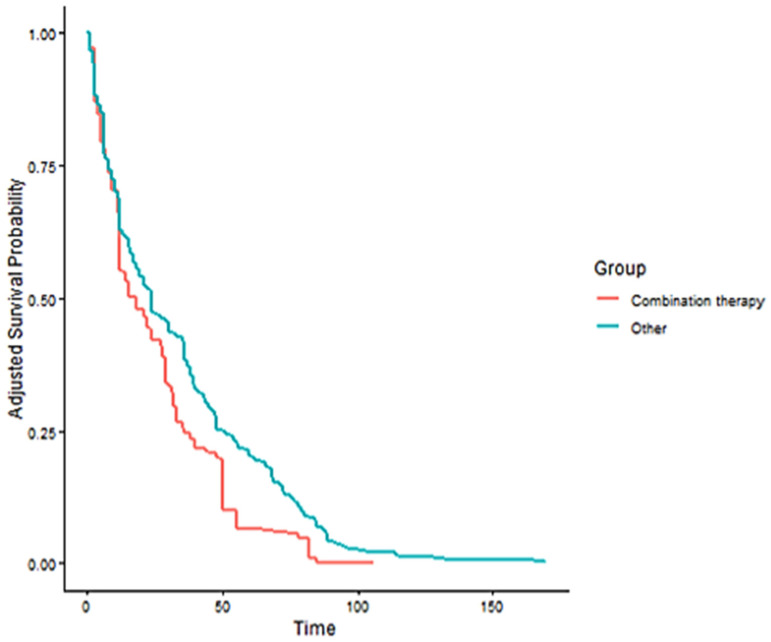
Kaplan–Meier estimates for primary patency after inverse probability of treatment weighting (IPTW) comparing patients treated with combined therapy (CT) and non-combined therapy.

After inverse probability of treatment weighting, baseline covariates were adequately balanced between the CT and non-CT groups, as shown in Figure S2. In the weighted analysis, overall survival did not differ significantly between groups, although a tendency toward worse outcomes was observed in the CT group (CT vs. non-CT: HR of 1.20, 95% CI of 0.92–1.59; log-rank test, *p* = 0.001; robust log-rank, *p* = 0.20; Figure 5). In contrast, CT was associated with significantly reduced primary patency compared with non-CT patients (CT vs. non-CT: HR of 1.37, 95% CI of 1.02–1.85; log-rank test, *p* < 0.0001; robust log-rank, *p* = 0.03; Figure 6). 

Adjusted hazard ratio: 1.20 (95% CI of 0.92–1.59); *p*-value for log-rank test, 0.001; *p*-value for robust log-rank test, 0.2.

Cox regression: adjusted hazard ratio: 1.37 (95% CI 1.02–1.85); *p*-value for log-rank test, <0.0001; *p*-value for robust log-rank test, 0.03.

## 4. Discussion

This subanalysis of the PARADE registry shows that overall survival, as well as primary and secondary patency rates, appear to be significantly worse in patients receiving a combination of oral anticoagulant and antiplatelet therapy after open popliteal artery aneurysm repair with posterior approach (OPAR). These findings were consistently confirmed across multivariable Cox regression, competing risk analysis, and inverse probability of treatment weighting, while no significant differences were observed among the other antithrombotic regimens. The validity of our treatment group comparisons is supported by the inverse probability of treatment weighting (IPTW) analysis, which demonstrated adequate balance of baseline covariates between the combined therapy group and all other antithrombotic regimens (Supplementary Figure S2). Competing-risk analysis using the Fine–Gray model revealed a significant difference in cumulative incidence of primary patency loss between antithrombotic regimen groups (*p* = 0.024), with no significant difference in the competing event of death (*p* = 0.635), suggesting that the observed patency differences are not confounded by differential mortality (Supplementary Figure S1). Notably, multivariable regression identified symptomatic clinical presentation as the only independent predictor of 30-day major adverse cardiovascular events (OR of 3.76, 95% CI of 1.16–12.18, *p* = 0.027), while antithrombotic regimen type was not independently associated with early MACEs (Supplementary Table S1).

Postoperative medical management after OPAR remains a partially investigated field, and available evidence is scarce and largely extrapolated from studies on peripheral arterial occlusive disease. In our cohort, only a small proportion of patients were not receiving any antithrombotic therapy preoperatively, reflecting the high prevalence of cardiovascular comorbidities, including coronary artery disease and atrial fibrillation.

Early outcomes after OPAR were satisfactory, with high technical success and low perioperative mortality, in line with previous reports. Although early graft occlusions were not uncommon, they were generally amenable to prompt reintervention. Moreover, previous studies comparing autologous vein and prosthetic conduits did not demonstrate significant differences in early outcomes, suggesting that early graft performance is primarily influenced by technical and procedural factors rather than postoperative medical therapy alone [20].

Consistently, postoperative antithrombotic therapy showed no significant association with 30-day primary patency or major adverse cardiovascular events. This finding is likely explained by the relatively low early event rate and by the predominant role of perioperative management and baseline patient characteristics in the immediate postoperative period. Nevertheless, the absence of early benefit should be interpreted cautiously, as the short follow-up may be insufficient to capture the long-term effects of pharmacological strategies.

At long-term follow-up, overall survival was as high as 90.3%, which is also consistent with the relatively low mean age of the cohort. Importantly, no differences were found between VKA and DOAC, nor between SAPT or DAPT and anticoagulation alone. A similar pattern was observed for both primary and secondary patency, which were significantly reduced in patients receiving CT. Although a direct correlation between CT and graft durability was observed, these findings should be interpreted with caution, as alternative explanations for the association remain possible. CT may identify a subgroup of patients with advanced systemic cardiovascular disease, higher thrombo-inflammatory burden, and increased frailty. Despite comprehensive multivariable adjustment, competing risk analysis, and inverse probability of treatment weighting showing a good balance of all recorded variables (Figure S2), residual confounding remains possible. Patients treated with CT are more likely to have atrial fibrillation, established coronary artery disease, diffuse atherosclerosis, and systemic endothelial dysfunction, the cumulative impact of which may not be fully accounted for by the recorded baseline variables. Importantly, the PARADE registry did not systematically record the indication underlying postoperative antithrombotic therapy selection. Consequently, treatment allocation likely reflected physician assessment of individual thrombotic and cardiovascular risk profiles, limiting causal interpretation of the observed associations. Among the 113 patients discharged on combined therapy (CT), preoperative treatment patterns were heterogeneous: 57 patients (50.4%) were receiving SAPT, 41 (36.3%) were already on CT, 9 (8.0%) were treated with oral anticoagulation alone, 2 (1.8%) were on DAPT, and 4 (3.5%) were not receiving any antithrombotic treatment. While these data provide additional insight into baseline treatment patterns within this subgroup, they do not clarify the clinical rationale for escalation to or continuation of combined therapy after aneurysm repair.

From a biological standpoint, while possibly speculative, excessive platelet inhibition combined with systemic anticoagulation may interfere with vascular healing processes. Platelet-mediated signaling plays a role in endothelial regeneration and graft remodeling, and over-suppression of this pathway could theoretically impair endothelialization and favor late graft dysfunction, particularly in aneurysmal disease, where the thrombotic milieu differs from that of occlusive PAD. Furthermore, combined therapy is associated with an increased risk of bleeding, including subclinical bleeding and chronic anemia, which may negatively impact physiological reserve and long-term survival. Although bleeding complications were not systematically recorded in the PARADE registry, their indirect contribution to mortality, reduced graft surveillance, and lower reintervention rates cannot be excluded.

When contextualized within the existing literature, these findings are consistent with previous reports. A recent analysis comparing great saphenous vein and ePTFE grafts in elective posterior PAA repair showed worse patency in prosthetic grafts among patients not receiving antithrombotic therapy, suggesting a protective role of antithrombotic agents in selected settings [21]. However, that study did not evaluate combined therapy specifically. Conversely, Kim et al. demonstrated that no combination of antiplatelet and anticoagulant therapy was associated with improved graft patency following femoropopliteal bypass for occlusive PAD [22]. Large randomized trials in PAD populations, such as COMPASS and VOYAGER-PAD, showed modest reductions in ischemic events with intensified antithrombotic regimens at the expense of increased bleeding risk [9,10]. However, these trials did not include patients undergoing OPAR, strongly limiting extrapolation to the PAA population.

Overall, although CT remained independently associated with graft failure after adjustment in the present study, the finding may also capture residual aspects of patient frailty and comorbidity not fully accounted for in the model. This interpretation aligns with current European and North American guidelines, which recommend SAPT after open popliteal aneurysm repair, reserving combined regimens for patients with compelling systemic indications [7,8]. Importantly, the results from the study do not support the addition of adjunctive antiplatelet therapy in patients undergoing OPAR who are receiving preoperative anticoagulation for established comorbid conditions. Likewise, they do not provide evidence to support initiating anticoagulation after OPAR in patients already treated with antiplatelet therapy to improve graft patency.

### Limitations

This study has several limitations. First, its retrospective observational design is inherently susceptible to selection bias and confounding, and causal inferences cannot be drawn from the observed associations. In particular, confounding by indication remains a major concern, as patients receiving combined anticoagulant and antiplatelet therapy likely represented a higher-risk subgroup with greater cardiovascular and thrombotic burden. Although multivariable adjustment and inverse probability of treatment weighting achieved good balance across measured covariates, important factors influencing treatment allocation—such as atrial fibrillation, heart failure, prior cerebrovascular events, left ventricular dysfunction, hypercoagulable states, and physician-specific treatment decisions—were not systematically recorded in the PARADE registry. Consequently, residual confounding from unmeasured variables cannot be excluded.

Second, the relatively small size of some treatment subgroups, together with the heterogeneity of antithrombotic regimens and treatment practices across participating centers, may have limited statistical power and reduced the generalizability of the findings. Furthermore, insufficient sample sizes precluded robust comparisons between specific anticoagulant and antiplatelet drug combinations.

Third, bleeding complications were not systematically captured, preventing assessment of the balance between thrombotic protection and bleeding risk associated with different antithrombotic strategies. Similarly, detailed information on relevant cardiovascular risk factors, including statin therapy and smoking exposure, was not uniformly available across centers.

Finally, postoperative imaging surveillance was not standardized and reflected local clinical practice, potentially introducing variability in event detection and follow-up.

## 5. Conclusions

The optimal postoperative antithrombotic regimen after open popliteal artery aneurysm repair remains uncertain. Postoperative medical therapy is not associated with 30-day primary patency or major adverse cardiovascular events. However, during long-term follow-up, overall survival and both primary and secondary patency appear to be significantly worse in patients receiving combined oral anticoagulant and antiplatelet therapy. Even if these findings may also reflect an undetected elevated baseline cardiovascular risk rather than exclusively a direct adverse effect of combined therapy, our findings do not support the routine use of combined anticoagulant and antiplatelet therapy after open popliteal artery aneurysm repair for the sole purpose of improving graft patency, and this combination should be reserved for patients with a compelling systemic indication. A major limitation of this study is the lack of data on bleeding complications, which were not recorded in the registry. As bleeding may itself contribute to the worse long-term survival and patency observed with combined therapy, the absence of these data limits the interpretation of our findings and should be taken into account when considering them. Given the observational design of this study, residual confounding by indication may still account for part of the observed associations, as patients receiving combined therapy likely represented a higher-risk subgroup; these findings should therefore be interpreted as hypothesis-generating rather than causal. Further prospective studies are needed to better define individualized postoperative antithrombotic strategies in this patient population.

## Figures and Tables

**Table 1:** Baseline and demographic data.

Characteristic	N = 638 a
Age	70 [64–77]
Age > 80	112 (17.6)
Male	615 (96.4)
Clinical presentation	
Asymptomatic	436 (68.3)
Intermittent claudication	115 (18.0)
CLTI	87 (13.6)
Smoke	
Current	235 (36.8)
Former	216 (33.9)
None	187 (29.3)
Hypertension	482 (75.5)
Hyperlipidaemia	384 (60.3)
Diabetes	118 (18.5)
Insulin treatment	31 (4.9)
Oral hypoglycemic agents	87 (13.6)
CAD history	178 (27.9)
CKD (GFR < 30 mL/min)	26 (4.1)
ESKD	4 (0.6)
Pre-operative therapy	
No therapy	72 (11.3)
SAPT	404 (63.3)
DAPT	40 (6.3)
VKA	32 (5.0)
DOAC	41 (6.4)
VKA/DOAC + SAPT	49 (7.7)

Abbreviations: CAD, coronary artery disease; CKD, chronic kidney disease; CLTI, Chronic Limb Threatening Ischemia; ESKD, end-stage kidney disease; DAPT, double antiplatelet therapy; DOAC, direct oral anticoagulant therapy; SAPT, single antiplatelet therapy; VKA, vitamin K antagonist. a Data are presented as count (%) or median [interquartile range].

**Table 2:** Intraoperative details.

Characteristic	N = 638 a
Graft material b	
Autologous vein	296 (46.6)
Prosthetic graft	322 (50.7)
Homograft	15 (2.4)
Missing data	5 (0.8)
In Autologous vein group b	296
Ipsilateral great saphenous vein	240 (81.8)
Ipsilateral small saphenous vein	26 (8.8)
Contralateral great saphenous vein	12 (4.1)
Contralateral small saphenous vein	1 (0.3)
Arm vein	13 (4.4)
Missing data	4 (1.3)
In Prosthetic graft group c	322
ePTFE	266 (82.6)
Dacron	42 (13.0)
Omniflow	7 (2.4)
Fusion graft	6 (1.9)
Missing data	1 (0.3)
Bypass length (mm)	65 [50–100]
Graft diameter (mm)	7.00 [5.00–8.00]
Runoff vessel	
0	5 (0.8)
1	94 (14.7)
2	214 (33.5)
3	325 (50.9)
Adjunctive procedures d	55 (8.6)
BTK thrombectomy	32 (58.2)
BTK PTA	7 (12.7)
SFA endoarterectomy	3 (5.4)
SFA stenting	2 (3.6)
SFA PTA	4 (7.3)
Thrombolysis	1 (1.8)
Muscle resection	4 (7.3)
Minor amputation	1 (1.8)
Iliac stenting	1 (1.8)
Technical success	633 (99.4)

Abbreviations: ePTFE, expanded polytetrauoroethylene; a data are presented as count (%) or median [interquartile range]; percentage in column, when not otherwise specified. b Percentages are computed based on the number of autologous vein bypasses; c percentages are computed based on the number of prosthetic graft bypasses; d percentages are computed based on the number of adjunctive procedures.

**Table 3:** Post-operative antithrombotic therapy.

Characteristic	N = 638
SAPT	359 (56.3)
ASA	290 (45.5)
Clopidogrel	62 (9.7)
Dipyridamole	3 (0.5)
Ticagrelor	3 (0.5)
DAPT	92 (14.4)
ASA + Plavix	89 (13.9)
ASA + Ticagrelor	3 (0.5)
Anticoagulant	74 (11.6)
VKA	35 (5.5)
DOAC	39 (6.1)
CT (Anticoagulant + SAPT)	113 (17.7)
DOAC + ASA	48 (7.5)
DOAC + Plavix	4 (0.63)
VKA + ASA	57 (8.9)
VKA + Plavix	2 (0.31)
DAPT + DOAC	2 (0.31)

Abbreviations: CT, combined therapy; ASA, acetylsalicylic acid; DAPT, double antiplatelet therapy; DOAC, direct oral anticoagulant; SAPT, single antiplatelet therapy; VKA, vitamin K antagonist.

**Table 4:** Univariable and multivariable Cox model for overall survival.

Univariable Analysis			Multivariable Analysis	
Variable	HR (95% CI)	*p*-Value	HR (95% CI)	*p*-Value
Post-operative therapy (CT vs. others)	1.30 (1.04–1.61)	0.018	1.30 (1.04–1.61)	0.018
Pre-OP therapy (SAPT vs. no therapy)	1.06 (0.82–1.37)	0.664	1.06 (0.82–1.37)	0.664
Pre-OP therapy (DAPT vs. no therapy)	1.52 (1.02–2.26)	0.038	1.52 (1.02–2.26)	0.058
Pre-OP therapy (VKA vs. no therapy)	0.75 (0.47–1.20)	0.233	0.75 (0.47–1.20)	0.233
Pre-OP therapy (DOAC vs. no therapy)	1.18 (0.80–1.75)	0.410	1.18 (0.80–1.75)	0.410
Pre-OP therapy (CT vs. no therapy)	1.27 (0.86–1.85)	0.226	1.27 (0.86–1.85)	0.226
Graft (vein vs. other)	1.11 (0.94–1.31)	0.207	1.11 (0.94–1.31)	0.207
Bypass length (25–50 mm vs. <25 mm)	1.42 (0.92–2.19)	0.111	1.42 (0.92–2.19)	0.111
Bypass length (50–75 mm vs. <25 mm)	0.86 (0.59–1.26)	0.448	0.86 (0.59–1.26)	0.448
Bypass length (>=75 mm vs. <25 mm)	1.27 (0.96–1.68)	0.092	1.27 (0.96–1.68)	0.092
Bypass length (Unknown vs. <25 mm)	1.18 (0.97–1.43)	0.101	1.18 (0.97–1.43)	0.101
Runoff vessels	1.04 (0.94–1.16)	0.462	1.04 (0.94–1.16)	0.462

Abbreviations: CT, combined therapy (anticoagulant + SAPT); DAPT, double antiplatelet therapy; DOAC, direct oral anticoagulant; SAPT, single antiplatelet therapy; VKA, vitamin K antagonist. Note: The reference group is always the one at the end; e.g., for A vs. B the reference group is B. For the multivariable model: C = 0.564, AIC = 6385.

**Table 5:** Univariable and multivariable Cox model for primary patency.

Univariable Analysis			Multivariable Analysis	
Variable	HR (95% CI)	*p*-Value	HR (95% CI)	*p*-Value
Post-operative therapy (CT vs. others)	1.47 (1.14–1.92)	<0.001	1.47 (1.14–1.92)	0.004
Pre-OP therapy (SAPT vs. no therapy)	1.22 (0.93–1.59)	0.150	1.18 (0.89–1.56)	0.241
Pre-OP therapy (DAPT vs. no therapy)	1.54 (1.02–2.35)	0.041	1.48 (0.97–2.28)	0.071
Pre-OP therapy (VKA vs. no therapy)	1.16 (0.74–1.82)	0.524	1.14 (0.72–1.79)	0.586
Pre-OP therapy (DOAC vs. no therapy)	1.12 (0.72–1.75)	0.609	1.06 (0.68–1.67)	0.797
Pre-OP therapy (CT vs. no therapy)	1.47 (0.99–2.18)	0.053	1.06 (0.67–1.66)	0.816
Graft (Vein vs. other)	1.11 (0.94–1.31)	0.239	1.12 (0.94–1.33)	0.198
Bypass length (25–50 mm vs. <25 mm)	1.46 (0.92–2.31)	0.105	1.44 (0.91–2.29)	0.120
Bypass length (50–75 mm vs. <25 mm)	0.71 (0.48–1.05)	0.088	0.70 (0.47–1.04)	0.077
Bypass length (>=75 mm vs. <25 mm)	1.39 (1.04–1.86)	0.027	1.41 (1.05–1.90)	0.023
Bypass length (Unknown vs. <25 mm)	1.00 (0.82–1.22)	0.992	1.00 (0.81–1.23)	0.987
Runoff vessels	1.01 (0.91–1.12)	0.895	1.01 (0.90–1.13)	0.912

Abbreviations: CT, combined therapy (anticoagulant + SAPT); DAPT, double antiplatelet therapy; DOAC, direct oral anticoagulant; SAPT, single antiplatelet therapy; VKA, vitamin K antagonist. Note: The reference group is always the one at the end; e.g., for A vs. B the reference group is B. For the multivariable model: C = 0.572, AIC = 5944.

**Table 6:** Univariable and multivariable Cox model for secondary patency.

Univariable Analysis			Multivariable Analysis	
Variable	HR (95% CI)	*p*-Value	HR (95% CI)	*p*-Value
Post-operative therapy (CT vs. others)	1.35 (1.10–1.69)	0.003	1.35 (1.10–1.69)	0.018
Pre-OP therapy (SAPT vs. no therapy)	1.16 (0.90–1.49)	0.266	1.12 (0.86–1.45)	0.411
Pre-OP therapy (DAPT vs. no therapy)	1.58 (1.06–2.34)	0.024	1.48 (0.99–2.21)	0.057
Pre-OP therapy (VKA vs. no therapy)	1.05 (0.69–1.60)	0.828	0.99 (0.64–1.52)	0.962
Pre-OP therapy (DOAC vs. no therapy)	1.21 (0.80–1.83)	0.373	1.13 (0.74–1.73)	0.560
Pre-OP therapy (CT vs. no therapy)	1.47 (1.01–2.13)	0.043	1.15 (0.75–1.77)	0.521
Graft (Vein vs. other)	1.16 (0.99–1.36)	0.073	1.18 (1.00–1.40)	0.045
Bypass length (25–50 mm vs. <25 mm)	1.54 (0.97–2.43)	0.065	1.55 (0.98–2.46)	0.062
Bypass length (50–75 mm vs. <25 mm)	0.72 (0.48–1.06)	0.098	0.70 (0.47–1.05)	0.085
Bypass length (>=75 mm vs. <25 mm)	1.30 (0.98–1.73)	0.067	1.33 (0.99–1.78)	0.059
Bypass length (Unknown vs. <25 mm)	1.07 (0.89–1.30)	0.472	1.08 (0.89–1.32)	0.425
Runoff vessels	0.98 (0.89–1.09)	0.726	0.98 (0.88–1.09)	0.730

Abbreviations: CT, combined therapy (anticoagulant + SAPT); DAPT, double antiplatelet therapy; DOAC, direct oral anticoagulant; SAPT, single antiplatelet therapy; VKA, vitamin K antagonist. Note: The reference group is always the one at the end; e.g., for A vs. B the reference group is B. For the multivariable model: C = 0.564, AIC = 6386.

## Data Availability

Aggregate data are available upon reasonable request.

## Supplementary Materials

The following supporting information can be downloaded at https://www.mdpi.com/article/10.3390/jcm15145364/s1. Figure S1: Cumulative incidence curves for loss of primary patency according to antithrombotic regimen, accounting for death as a competing risk using the Fine–Gray model. A significant difference in the cumulative incidence of primary patency loss was observed between treatment groups (*p* = 0.024), whereas no significant differences were found for the competing event of death (*p* = 0.635). Figure S2: Plot of covariates balance for clinically significant variables in group of patients under post-operative combined therapy vs. all others. The orange dot represents the unadjusted variables distribution and the light blue one the mean difference after the balance. Table S1: Multivariable logistic regression model for 30-day MACEs. Table S2: Univariable and multivariable logistic regression model for 30-day primary patency and re-interventions. Table S3: Deaths and causes. Table S4: Baseline comparison of patients under combiner therapy with other. Supplementary S1: PARADE Study Collaborative Group (all citable on PUBMED).

## Abbreviations

The following abbreviations are used in this manuscript:

PAA	Popliteal artery aneurysms
MACE	Major adverse cardiovascular event
VKA	Vitamin K antagonist
DOAC	Direct oral anticoagulant
PAD	Peripheral arterial disease
RCPAD	Research Collaborative in Peripheral Arterial Disease
PARADE	Popliteal ARtery Aneurysms with posterior approach vs. enDovascular Exclusion
DUS	Duplex ultrasound
CTA	Computed tomography angiography
MI	Myocardial infarction
SAPT	Single antiplatelet therapy
DAPT	Dual antiplatelet therapy
SD	Standard deviation
IQR	Interquartile range
HR	Hazard ratio
CI	Confidence interval
CAD	Coronary artery disease
CKD	Chronic kidney disease
CLTI	Chronic Limb Threatening Ischemia
ESKD	End-stage kidney disease
ePTFE	Expanded polytetrauoroethylene
ASA	Acetylsalicylic acid
CT	Combined therapy
IPTW	Inverse probability of treatment weighting
OPAR	Open popliteal artery aneurysm repair
