# Supplementary material for: Impact of Postoperative Antithrombotic Treatment on Patency and Survival After Open Posterior Popliteal Artery Aneurysm Repair: A Multicenter Retrospective Cohort Analysis from the PARADE Study
Source: J Clin Med. 2026 Jul 9;15(14):5364. doi: 10.3390/jcm15145364 (PMC13410262; doi:10.3390/jcm15145364)
Supplement: Supplementary file 1 [file jcm-15-05364-s001.zip › jcm-4335763-supplementary.pdf]

Supplementary Materials

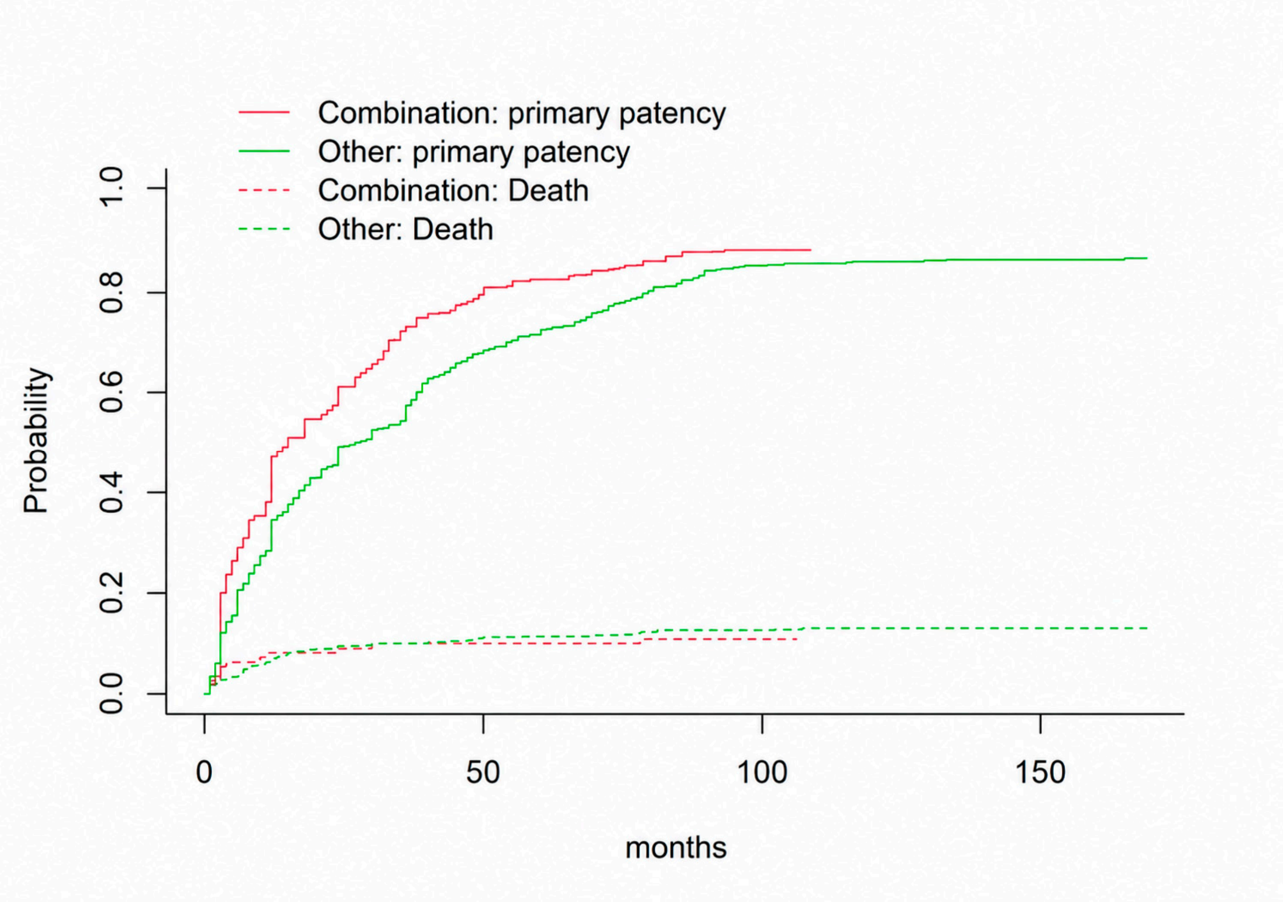

Supplementary Figure S1: Cumulative incidence curves for loss of primary patency according to antithrombotic regimen, accounting for death as a competing risk using the Fine–Gray model. A significant difference in the cumulative incidence of primary patency loss was observed between treatment groups (p = .024), whereas no significant differences were found for the competing event of death (p = .635).

Abbreviations: CT, combined therapy

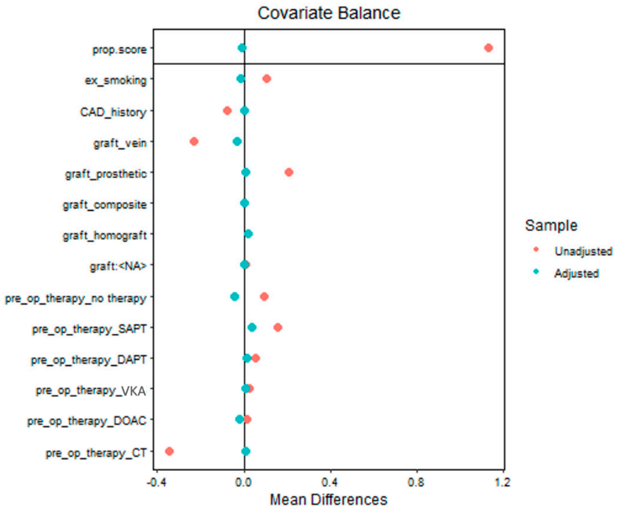

Supplementary Figure S2: Plot of covariates balance for clinically significant variables in group of patients under post-operative combined therapy vs all others. The orange dot represents the unadjusted variables distribution and the light blue one the mean difference after the balance.

Abbreviations: CT, combined therapy; DAPT, double antiplatelet therapy; DOAC, direct oral anticoagulant; N/A, not available data; VKA, vitamin K antagonist; SAPT, single antiplatelet therapy;

Supplementary Table S1: Multivariable logistic regression model for 30-day MACE.

**Univariable model**			**Multivariable model**	
**MACE**				
**Variable**	**Odds ratio (95% CI)**	**p-value**	**Odds ratio (95% CI)**	**p-value**
Clinical presentation symptomatic	5.40 (1.87 – 15.59)	0.002	3.76 (1.16 - 12.18)	0.027
Diabetes	6.02 (1.32 – 27.28)	0.020	3.85 (0.61 - 24.27)	0.151
CAD history	15.94 (1.90 – 133.37)	0.011	8.95 (0.90 - 88.50)	0.061
Dialysis	34.77 (3.14 – 384.02)	0.004	4.89 (0.05 – 420.34)	0.479
Graft material	0.97 (0.29-3.23)	0.961		
Bypass lenght	0.98 (0.96-1.01)	0.322		
Post-operative therapy	0.74 (0.35-1.56)	0.436		
Post-operative Therapy (DAPT vs.SAPT)	16.22 (1.79 – 146.99)	0.643	6.36 (0.53 – 76.23)	0.144
Only 1 Runoff BTK vessel	14.72 (1.6-133.43	0.017	6.35 (0.48 – 83.39)	0.159

Abbreviations: BTK, below the knee; CAD, coronary artery disease; DAPT, double antiplatelet therapy; SAPT, single antiplatelet therapy.

Supplementary Table S2: Univariable and multivariable logistic regression model for 30-day primary patency and re-interventions.

**Univariable model**			**Multivariable model**	
30-day primary patency failure				
Variable	Odds ratio (95% CI)	p-value	Odds ratio (95% CI)	p-value
Pre-OP therapy (SAPT vs. no therapy)	1.08 (0.13 - 9.13)	0.942	1.12 (0.13 - 9.72)	0.919
Pre-OP therapy (DAPT vs. no therapy)	3.68 (0.32 - 41.96)	0.293	3.67 (0.31 - 43.80)	0.303
Pre-OP therapy (DOAC vs. no therapy)	3.59 (0.32 - 40.86)	0.303	3.71 (0.32 - 43.12)	0.295
Pre-OP therapy (CT vs. no therapy)	2.98 (0.26 - 33.79)	0.378	2.69 (0.17 - 41.81)	0.479
Therapy (Other vs. Combination therapy)	0.73 (0.20 - 2.71)	0.643	0.84 (0.16 - 4.45)	0.838
Graft (Vein vs. Other)	1.41 (0.47 - 4.25)	0.540	1.51 (0.49 - 4.66)	0.477
Bypass length (50-75 vs. 25-50)	0.48 (0.12 - 1.89)	0.295	0.47 (0.12 - 1.89)	0.286

Univariable model			Multivariable model	
30-day primary patency failure				
Variable	Odds ratio (95% CI)	p-value	Odds ratio (95% CI)	p-value
Bypass length (>=75 vs. 25-50)	0.64 (0.19 - 2.10)	0.460	0.63 (0.19 - 2.12)	0.454
Bypass length (Unknown vs. 25-50)	0.94 (0.35 - 2.55)	0.903	0.92 (0.33 - 2.57)	0.872
Runoff vessels	0.92 (0.45 - 1.90)	0.825	0.94 (0.45 - 1.97)	0.880
30 day re-intervention				
Pre-OP therapy (SAPT vs. no therapy)	0.99 (0.21 - 4.57)	0.991	0.93 (0.20 - 4.43)	0.930
Pre-OP therapy (DAPT vs. no therapy)	1.82 (0.25 - 13.41)	0.559	1.84 (0.24 - 13.95)	0.556
Pre-OP therapy (VKA vs. no therapy)	2.30 (0.31 - 17.10)	0.416	2.17 (0.29 - 16.40)	0.453
Pre-OP therapy (DOAC vs. no therapy)	2.72 (0.44 - 17.02)	0.284	2.64 (0.41 - 16.89)	0.307
Pre-OP therapy (CT vs. no therapy)	1.47 (0.20 - 10.79)	0.706	0.88 (0.10 - 7.86)	0.910
Therapy (Other vs. Combination therapy)	0.56 (0.21 - 1.46)	0.237	0.52 (0.17 - 1.62)	0.262
Graft (Vein vs. Other)	1.44 (0.61 - 3.38)	0.406	1.27 (0.52 - 3.06)	0.602
Bypass length (50-75 vs. 25-50)	1.34 (0.45 - 3.98)	0.594	1.36 (0.44 - 4.14)	0.591
Bypass length (>=75 vs. 25-50)	0.98 (0.37 - 2.59)	0.966	0.94 (0.35 - 2.53)	0.901
Bypass length (Unknown vs. 25-50)	1.10 (0.47 - 2.58)	0.820	1.14 (0.48 - 2.72)	0.771
Runoff vessels	0.82 (0.48 - 1.41)	0.473	0.80 (0.46 - 1.40)	0.430

Abbreviations: CT, combined therapy; DAPT, double antiplatelet therapy; DOAC, direct oral anticoagulant;; SAPT, single antiplatelet therapy, VKA, Vitamin K antagonist

Supplementary Table S3: Deaths causes.

Characteristics	Data
Total deaths	62 (9.7%)b
Acute MI	9 (14.5%)c

Cancer	9 (14.5%)c
COPD	1 (1.6%)c
COVID-19	2 (3.2%)c
ESRD	1 (1.6%)c
Fatal arrhytmia	3 (4.8%)c
Head injury	1 (1.6%)c
Heart failure	3 (4.8%)c
MOF	2 (3.2%)c
Pneumonia	1 (1.6%)c
Pulmonary edema	2 (3.2%)c
Ruptured AAA	1 (1.6%)c
Sepsis	3 (4.8%)c
Stroke	3 (4.8%)c
Unknown	21 (33.9%)c

Abbreviations: AAA, abdominal aortic aneurysm; COPD, congestive obstructive pulmonary disease; ESRD, end stage renal disease; MI, myocardial infarction; MOF, multiple organ failure.

a Data are presented as count (%)
b Percetges computed on the total number of patients
c Percentages computed on the number of deaths

Supplementary figure I: Cumulative incidence curves for loss of primary patency according to antithrombotic regimen, accounting for death as a competing risk using the Fine–Gray model. A significant difference in the cumulative incidence of primary patency loss was observed between treatment groups (p = 0.024), whereas no significant differences were found for the competing event of death (p = 0.635).

Supplementary Table S4: Baseline comparison of patients under combiner therapy with other

**Characteristic**	**Combined therapy**[a, b] N = 111	**Other**[a, b] N = 527	**p-value**
age	70 (64, 79)	70 (63, 77)	0.7
age>80 (0=no, 1=yes)	24 (21.6%)	88 (16.7%)	0.2
sex (m, f)			0.4
f	2 (1.8%)	21 (4.0%)	
m	109 (98.2%)	506 (96.0%)	
Clinical presentation (0=asymptomatic, 1=intermittent claudication, 2=CLTI)			0.7
0	77 (69.4%)	359 (68.1%)	
1	17 (15.3%)	98 (18.6%)	
2	17 (15.3%)	70 (13.3%)	
smoking	38 (34.2%)	197 (37.4%)	0.5

ex smoking	28 (25.2%)	188 (35.7%)	0.034
hypertension	78 (70.3%)	404 (76.7%)	0.2
hyperlypidaemia	74 (66.7%)	310 (58.9%)	0.13
Unknown	0	1	
diabetes	15 (13.5%)	103 (19.5%)	0.14
insulin treatment	3 (2.7%)	28 (5.3%)	0.2
CAD history	38 (34.2%)	140 (26.6%)	0.10
CKD (GFR<30ml/min)	2 (1.8%)	24 (4.6%)	0.3
dialysis	0 (0.0%)	4 (0.8%)	>0.9
Graft material (1=vein, 2=prosthetic, 3=composite, 4=homograft)			<0.001
1	73 (65.8%)	223 (42.6%)	
2	37 (33.3%)	285 (54.4%)	
3	0 (0.0%)	2 (0.4%)	
4	1 (0.9%)	14 (2.7%)	
Unknown	0	3	
If vein (1=great saphenous vein, 2=small saphenous vein, 3=contralateral great saphenous vein, 4= contrateral small saphenous vein, 5=arm vein, 6=other)			0.3
1	63 (86.3%)	177 (80.1%)	
2	3 (4.1%)	23 (10.4%)	
3	5 (6.8%)	7 (3.2%)	
4	0 (0.0%)	1 (0.5%)	
5	2 (2.7%)	11 (5.0%)	
6	0 (0.0%)	2 (0.9%)	
Unknown	38	306	
If prosthetic (1=ePTFE, 2=Dacron, 3=Bovine pericardium, 4=Omniflow, 5=Hybrid, 6=Fusion)			>0.9
1	32 (86.5%)	234 (81.8%)	
2	4 (10.8%)	38 (13.3%)	
4	1 (2.7%)	6 (2.1%)	
5	0 (0.0%)	2 (0.7%)	
6	0 (0.0%)	6 (2.1%)	
Unknown	74	241	
Bypass length (mm)	73 (55, 110)	63 (50, 100)	0.10
Unknown	47	149	
Graft diameter (mm)	7.00 (4.50, 8.00)	7.00 (5.00, 8.00)	0.5
Unknown	45	72	
Adjunctive procedures (0=no, 1=yes)	9 (8.1%)	47 (9.0%)	0.8
Unknown	0	2	
Acute technical success (0=no, 1=yes)			0.14
0	2 (1.8%)	2 (0.4%)	
1	109 (98.2%)	524 (99.6%)	
Unknown	0	1	

mortality (0=no, 1=yes)	1 (0.9%)	0 (0.0%)	0.2
MACE (0=no, 1=yes)	2 (1.8%)	5 (1.0%)	0.3
Unknown	1	1	
graft occlusion (0=no, 1=yes)	3 (2.7%)	10 (1.9%)	0.5
reintervention (0=no, 1=yes)	6 (5.4%)	16 (3.0%)	0.2
Med therapy total (1= none, 2 = ASA, 3= Clopidogrel; 4= DAPT; 5 other; 6=OAC; 7=DOAC; 8=DOAC+ASA; 9 DOAC+Plavix; 10 OAC+ASA; 11 OAC+Plavix; 12 triplice terapia)			<0.001
2	0 (0.0%)	290 (55.0%)	
3	0 (0.0%)	62 (11.8%)	
4	0 (0.0%)	92 (17.5%)	
5	0 (0.0%)	7 (1.3%)	
6	0 (0.0%)	35 (6.6%)	
7	0 (0.0%)	39 (7.4%)	
8	48 (43.2%)	0 (0.0%)	
9	4 (3.6%)	0 (0.0%)	
10	57 (51.4%)	0 (0.0%)	
11	2 (1.8%)	0 (0.0%)	
12	0 (0.0%)	2 (0.4%)	
survival (0=no, 1=yes)	100 (90.1%)	476 (90.3%)	>0.9
primary patency (0=no, 1=yes)	96 (86.5%)	454 (86.1%)	>0.9
secondary patency (0=no, 1=yes)	106 (95.5%)	498 (94.5%)	0.7
limb salvage (0=no, 1=yes)	110 (99.1%)	525 (99.6%)	0.4
reintervention (0=no, 1=yes)	11 (9.9%)	57 (10.8%)	0.8
run-off BTK (1, 2, 3)			0.5
0	0 (0.0%)	5 (0.9%)	
1	16 (14.4%)	78 (14.8%)	
2	32 (28.8%)	182 (34.5%)	
3	63 (56.8%)	262 (49.7%)	
Follow-up, months	24.9 (22.3 33.8)	26.3 (21.3 44.4)	0.2397
Follow-up Index	0.72 (0.45 0.95)	0.64 (0.38 0.9)	0.0627
a Data are presented as count (%) or Median [Interquartile range]<br>b Percentage on column, when not otherwise specified.			

Abbreviations: CAD, coronary artery disease; CKD, chronic kidney disease; CLTI, Chronic Limb Threatening Ischemia; ePTFE, expanded polytetrauoroethylene ESKD, end stage kidney disease; DAPT, double antiplatelet therapy; DOAC, direct oral anticoagulant therapy; SAPT, single antiplatelet therapy; VKA, vitamin K antagonist.

Supplementary S1:

PARADE Study Collaborative Group (all citable on PUBMED)

Daniele Adami, Marco Andreini
Vascular Surgery Unit, Department of Translational Research and New Technologies in Medicine and Surgery, University of Pisa, Italy

Raffaele Pulli, Walter Dorigo, Carlo Pratesi, Sara Speziali, Brigida Biancofiore
Section of Vascular Surgery, Department of Excellence of Experimental and Clinical Medicine, University of Florence, Florence, Italy

Franco Grego, Michele Antonello, Michele Piazza, Francesco Squizzato, Elda Chiara Colacchio
Vascular and Endovascular Surgery Division, Department of Cardiac, Thoracic, Vascular Sciences, and Public Health, Padova University, Padova, Italy

Stefano Bonardelli, Luca Bertoglio, Paolo Baggi, Apollonia Verrengia
Division of Vascular Surgery, Department of Surgical and Clinical Sciences, University of Brescia School of Medicine, ASST Spedali Civili of Brescia, Brescia, Italy

Arnaldo Ippoliti, Fabio Massimo Oddi, Andrea Ascoli Marchetti
Vascular Surgery Unit, Department of Biomedicine and Prevention, Tor Vergata University, Rome, Italy

Luca Di Marzo, Wassim Mansour, Alessia Di Girolamo, Antonio Marzano
Vascular and Endovascular Surgery Division, Department of General Surgery and Surgical Specialties, Policlinico Umberto I, Sapienza University of Rome, Italy

Drosos Kotelis, Christian Zielasek, Dimitrios D Papazoglou
Department of Vascular Surgery, Inselspital, Bern University Hospital, University of Bern, Switzerland

Reinhold Perkmann, Marco D Pipitone
Department of Vascular and Thoracic Surgery, Bolzano Regional Hospital, Bolzano, Italy

Maurizio Taurino, Pasqualino Sirignano, Elisa Romano
Department of Vascular and Endovascular Surgery, Sant'Andrea Hospital, La Sapienza University, Rome, Italy

Massimo Lenti, Giacomo Isernia, Gioele Simonte, Gianluigi Fino
Department of Vascular Surgery, Azienda Ospedaliera di Perugia, Perugia, Italy

Gian F Veraldi, Luca Mezzetto
Department of Vascular Surgery, University Hospital and Trust of Verona, University of Verona-School of Medicine, Verona, Italy

Domenico Angiletta, Sergio Zacà, Margot Ringold, Francesca Sodero
Department of Precision and Regenerative Medicine and Jonic Area (DiMePre-J), Vascular and Endovascular Surgery, University of Bari School of Medicine "Aldo Moro", Bari, Italy

Georgios Pitoulias, Dimitrios A Chatzelas
Division of Vascular Surgery, 2nd Department of Surgery, Faculty of Medicine, School of Health Sciences, Aristotle University of Thessaloniki, G. Gennimatas Hospital, Thessaloniki, Greece

Aaron Fargion, Luca Traina, Gladiol Zenunaj
Unit of Vascular and Endovascular Surgery, University Hospital of Ferrara, Italy

Manar Khashram, Nusr Ghamri, Thomas M Lovelock
Department of Surgery, University of Auckland, New Zealand, Department of Vascular and Endovascular Surgery, Waikato Hospital, New Zealand

Hany Zayed, Ayman Hamdy, Lukla Biasi, Patel Sanjay
Department of Vascular Surgery, Guy's and St Thomas' NHS Foundation Trust, St Thomas' Hospital, London, United Kingdom

Sandro Lepidi, Mario D'Oria, Giovanni Badalamenti
Division of Vascular and Endovascular Surgery, Cardiovascular Department, University Hospital of Trieste ASUGI, Trieste, Italy

Massimo G Ruggiero, Claudio Desantis
Endovascular Surgery-Vascular Surgery Unit, ASL BR1-Ospedale A. Perrino, Brindisi, Italy

Federico Filippi, Manfredi Anzaldi
Department of Vascular Surgery, Hospital Misericordia, Grosseto, Italy

Andrea Siani, Federico Accrocca, Roberto Gabrielli, Stefano Bartoli
Unit of Vascular, Endovascular and Emergency Vascular Surgery, S. Eugenio Hospital, Rome, Italy

Massimiliano Martelli
Division of Vascular Surgery, MultiMedica Hospital, Sesto San Giovanni, Italy

Raimondo Micheli, Maria C Gugliotta
Unit of Vascular Surgery, Azienda Ospedaliera Santa Maria, Terni, Italy

Nikolaos Bessias, Konstantinos Roditis, Paraskevi Tsiantoula, Theofanis T Papas, Konstantinos G Seretis
Department of Vascular Surgery, Korgialenio-Benakio Hellenic Red Cross General Hospital, Athens, Greece

Marco V Usai
Department of Vascular Surgery, St Franziskus Hospital, Münster, Germany

Yamume Tshomba, Marco Natola, Lucia Scurto
Vascular Surgery Unit, Fondazione Policlinico Universitario A Gemelli IRCCS, Università Cattolica del Sacro Cuore, Rome, Italy

Grigorios Korosoglou, Christoph Schoefthaler, Amila Jehn
GRN Hospital Weinheim, Cardiology and Vascular Medicine, Weinheim, Germany

Athanasios Saratzis, Robert Davies
Department of Cardiovascular Sciences, University of Leicester, Glenfield Hospital, Leicester, United Kingdom

Gabriele Maritati
Vascular and Endovascular Unit, Ospedale dei Castelli - ASLRoma6, Rome, Italy

Shantonu Kumar Ghosh
National Institute of Cardiovascular Diseases (NICVD), Dhaka, Bangladesh

Konstantinos P Donas, Apostolos G Pitoulias, Konstantinos Avranas
Rhein Main Vascular Center, Department of Vascular and Endovascular Surgery, Asklepios Clinics Langen Wiesbaden, Seligenstadt, Germany

Gennaro Bafile
Vascular Surgery Unit, San Salvatore Hospital, University of L'Aquila, L'Aquila, Italy
Department of Vascular Surgery, SS Filippo e Nicola Hospital, Avezzano, Italy

Eugenio Martelli
Division of Vascular Surgery, Dept. of Biomedicine and Prevention, University of Rome Tor Vergata, Italy

Eduardo Cavallo
Division of Vascular Surgery, Department of Cardiovascular Sciences, S. Anna and S. Sebastiano Hospital, Caserta, Italy

Roberto Caronno, Aldo Alberti
Department of Vascular Surgery, Sant'Anna Hospital, Como, Italy

Pietro Volpe, Mafalda Massara, Antonino Alberti
Division of Vascular Surgery, Bianchi-Melacrino-Morelli Hospital, Reggio di Calabria, Italy

Gian F Fadda, Sara Zappadu
Unit of Vascular Surgery, Cliniche San Pietro Hospital, AOU Sassari, Sassari, Italy

Giovanni B Torsello
Institute for Vascular Research, St. Franziskus Hospital, Münster, Germany

Giuseppe Asciutto, Erika Lilja
Division of Vascular Surgery, Department of Surgical Sciences, Uppsala University, Uppsala, Sweden

Pablo del Canto Peruyera
Vascular and Endovascular Surgery Department. Cabueñes University Hospital, Gijón, Spain
